# The Treatment and Management of Oroantral Communications and Fistulas: A Systematic Review and Network Metanalysis

**DOI:** 10.3390/dj12050147

**Published:** 2024-05-20

**Authors:** Stefano Oliva, Felice Lorusso, Antonio Scarano, Maurizio D’Amario, Giovanna Murmura

**Affiliations:** 1 Department of Innovative Technologies in Medicine and Dentistry, University “G. d’Annunzio” of Chieti-Pescara, 66100 Chieti, Italy; felice.lorusso@unich.it (F.L.); a.scarano@unich.it (A.S.); g.murmura@unich.it (G.M.); 2 Department of Life, Health and Environmental Sciences, University of L’Aquila, 67100 L’Aquila, Italy; maurizio.damario@univaq.it

**Keywords:** oroantral communication, oroantral fistula, maxillary sinus, soft tissue local flaps, buccal fat pad

## Abstract

Objectives: The aim of this work was to systematically review and carry out a statistical metanalysis to identify the best treatment for close oroantral communications and fistulas and to avoid the risk of recurrence. Materials and Methods: An electronic search was conducted on the MEDLINE database (Pubmed), Scopus, and Google scholar using the following keywords: “oro antral communication (OAC)” OR “oro antral fistula (OAF)” OR “antro-oral communication” OR “communication between maxillary sinus and oral cavity” OR “oro-sinusal communication” OR “oro-sinusal fistula” OR “sinus communication” OR “sinus fistula” OR “antral communication” AND “treatment” OR “management” OR “surgical treatment” OR “surgical interventions”. This work was performed in accordance with the guidelines of PRISMA (Preferred Reporting Items for Systematic Reviews and Meta-analyses). After article screening, 9 RCTs (randomized controlled trials), comparing two or more techniques, were included in this review. Results: A statistically significant difference was detected in favor of the buccal fat pad compared to the buccal advancement flap and palatal rotational flap. Conclusions: With the limitations of this study, the buccal fat pad showed the best results in terms of communication closure and reducing the risk of relapse.

## 1. Introduction

The maxillary sinus is the largest paranasal sinus, and it usually occupies the entire body of the jawbone. It is lined on the inner side with a thin respiratory mucosa, which, on the medial side, is in continuity with the nasal cavity. The progressive expansion of the maxillary sinus, accompanied by a decrease in bone height in the posterior region of the maxillary bone, represents the risk of forming a link between the oral cavity and the maxillary sinus during oral surgery [1,2,3].

An oroantral communication (OAC) is a pathological pathway between the oral cavity and maxillary sinus that can lead to the appearance of several signs and symptoms, as well as infections of the maxillary sinus. Causes of an oroantral communication can be divided into iatrogenic and non-iatrogenic. An iatrogenic communication represents the majority. It can be caused by a technical mistake of the surgeon, or it can represent an inevitable event due to the anatomy of the maxillary sinus. An iatrogenic communication may result from the extraction of teeth with roots in relation to the maxillary sinus, the dislocation of teeth or roots in the maxillary sinus, the enucleation of periapical or cystic lesions whose walls adhere to the maxillary sinus mucosa, the preparation of implant sites in the lateral–posterior sectors of the maxilla, or the removal of neoplasms in the posterior sectors of the maxilla. Upper molar or premolar extraction is the most common cause of an OAC (48%) [4,5]. A non-iatrogenic communication is very uncommon and is a consequence of trauma that results in tooth avulsion or osteomyelitis. Defects less than 3 mm wide and without epithelialization may heal spontaneously in the absence of an infection, due to the organization of a primary blood clot [5,6]. Instead, in the presence of greater communication, the likelihood of spontaneous healing decreases, while the possibility of an infection, due to contamination of the oral microbial flora, increases. This may lead to the development of sinusitis [7]. In addition, bony defects > 3 mm require appropriate surgical treatment to close the communication and prevent a sinus infection and sinusitis. The timing of the treatment is a critical prognostic element; the literature indicates that the success rate in eliminating an OAC is 90 to 95 percent, if the treatment is started within 24 to 48 h. After 48 h, a sinus infection and oroantral fistulas are more likely to occur (OAFs) [8]. An oroantral fistula is an epithelialized OAC caused by the migration of oral epithelium into the defect. This epithelialization usually occurs when the perforation persists for at least 48–72 h, preventing a spontaneous closure of the perforation. After this period, it is not possible to effectively treat about half of affected patients due to increased inflammatory alterations [9]. The reported success rate for a secondary closure of OAFs has been as low as 67% [10]. Non treated OACs/OAFs resulted in 50% of sinusitis within 3 days and 90% after 2 weeks [11]. Clinically, small defects cannot be detected by inspection. The use of a small mirror or careful probing with a beveled tool may be helpful.

The most commonly reported symptoms include epistaxis, a leakage of fluid between the oral cavity and nose, pain, postnasal drip, altered vocal resonance, difficulty sucking, and puffiness of the cheeks. Symptoms vary widely and may occur after a prolonged period of time. Typical signs include muffled or increased sounds in the sinus during suction, the presence of small bubbles during the Valsalva maneuver, and the leakage of blood from the nose. Pain is usually absent, but it may be present in the malar region and may increase with a palpation of the anterior sinus wall [12]. OAF presence is characterized by the presence of an orifice from which there is a discharge of serous or purulent material. In larger defects, clinical presentation shows an antral polypoid herniation. A radiological examination is required to confirm the clinical findings. The size, location, and degree of sinus involvement can be determined with a panoramic radiograph and a CT scan [5,9,13]. Discontinuity of the sinus floor, sinus opacity, and focal alveolar atrophy are frequently observed. Computed tomography scans (CTs) and cone beam computed tomography (CBCT) scans are the gold standard for the radiologic evaluation of maxillary sinusitis [14]. Treatment options vary depending on the extension, epithelialization, and concomitant secondary sinus infection. Small communications, smaller than 3 mm, without an infection, usually heal spontaneously; clot formation leads to close communication. Surgical procedures are the treatment of choice for a defect size of >3–5 mm, in the presence of an infection.

Often, there is no infection after a perforation caused by the removal of a (pre)molar when closure is performed the same day. These kinds of perforations do not require previous pharmacological therapy.

A previous treatment of the sinus is required to reduce an infection, including the following:

- Antibiotics: a combination of antibiotics, such as amoxicillin and clavulanate potassium (625 mg), clindamycin (300 mg), 4 times daily, or moxifloxacin (400 mg), have been used in the treatment of OACs.
- Nasal decongestants: these can be used as adjuvants to heal OACs/OAFs, if the patient has any sinus infection 5.

These procedures can be divided into local flaps, distant flaps, and grafting [15].

Local flaps are usually used to close OACs/OAFs. Distant flaps and bone grafts are usually indicated for larger defects because of their larger tissue volumes.

### 1.1. Local Flaps

#### 1.1.1. Buccal Advancement Flap

This was described first by Rehrmann in 1936 [16].

The design of this flap involves two vertical incisions that diverge towards the buccal side, extending from the extraction socket or from the margins of the fistula orifice in the case of an OAF. The trapezoidal buccal flap is elevated and sutured over the defect at the palatal margins. Its wide base ensures adequate blood supply [17]. Von Wowern demonstrated that a decrease in the sulcus depth following the Rehrmann method persists in half of the reported cases [18].

#### 1.1.2. Buccal Pad of Fat Flap (BFP)

The anatomy of the buccal pad of the fat flap (BFP) was first described by Stajcic and later by Rapidis and Tideman. A 2–3 cm incision was made in the mucosa, positioned at least 2 cm below the Stensen’s duct. Subsequently, the buccinator and zygomaticus major muscles were incised, and a cautious blunt dissection was carried out to establish sufficient openings for the natural herniation of the fat pad, all the while avoiding damage to the capsule enveloping the fat pad [19].

This technique requires complete coverage by the oral mucosa.

According to the literature, it seems that an uncovered BFP typically goes through full epithelialization within a period of four to six weeks [20].

A rapid epithelialization of the exposed adipose tissue is a distinctive feature of the BFP flap pedicle and has been confirmed by histopathologic studies. The main disadvantage of this flap is the unpredictable restriction of the mouth opening. 

#### 1.1.3. Palatal Rotational Flap 

A full-thickness palatal flap is easily mobilized over the defect and is stronger and more resistant to infections and trauma. This technique has the advantage of a good blood supply through the palatal artery, rotations without tension, and the preservation of the buccal vestibule. However, the bony palatal surface is commonly exposed, causing pain and subsequent surface abnormalities in the surgical area due to a secondary epithelialization two or three months later [21,22]. For this reason, the palatal rotational flap is primarily used for defects close to the palatal site. A palatal splint or the use of sutures with collagen sponge material can be performed to improve the secondary epithelialization of the uncovered donor site.

#### 1.1.4. Platelet Rich Fibrin

In the last few decades, various authors have discussed the utilization of platelet-rich fibrin (PRF) as a substitute for these methods. PRF was introduced in 2001 by Choukroun et al. It represents the second generation of platelet concentrates that do not necessitate the addition of any platelet-activating agents (such as bovine thrombin or calcium chloride) [23].

A total of 30 to 40 mL of blood was collected from affected patients and was centrifuged (1500 rpm for 8 min). Three different layers were obtained. The top layer consisted of platelet-free plasma, the middle layer contained PRF, and the bottom layer contained erythrocytes. The isolated PRF was formed into a membrane. These membranes were placed in layers into the tooth socket so that they covered the OAC. Then, these membranes were fixed to the surrounding gingiva with the sutures. 

This systematic review aimed to evaluate the efficacy of different surgical flap treatments to repair oroantral communications and fistulas in terms of the success rate, the risk of recurrence, the number of complications, and patient morbidity (VAS score).

## 2. Materials and Methods

### 2.1. Search Strategy

This systematic review was performed in accordance with PRISMA (Preferred Reporting Items for Systematic Reviews and Meta-analyses) [24].

The review protocol was submitted to Prospero and registered with CRD42024514303 final registration number.

An electronic search was conducted on the MEDLINE database (Pubmed), Scopus, and Google scholar using the following keywords: “oro antral communication (OAC)” OR “oro antral fistula (OAF)” OR “antro-oral communication” OR “communication between maxillary sinus and oral cavity” OR “oro-sinusal communication” OR “oro-sinusal fistula” OR “sinus communication” OR “sinus fistula” OR “antral communication” AND “treatment” OR “management” OR “surgical treatment” OR “surgical interventions”. Only articles in the English language were considered, and no restrictions on date publication were applied. 

The titles and abstracts of the articles were subjected to an initial selection process considering relevance, the type of study, and the population considered.

### 2.2. Study Selection 

Two authors independently reviewed abstracts and titles located in the database. The controlled clinical trials of retrospective and prospective studies were selected for this systematic review.

The following inclusion criteria were applied:

- Studies reporting data on incidences and causes of OACs/OAFs;
- Studies in which an OAC/OAF was treated with one of these different surgical treatments: BFP, buccal advancement flap, palatal flap, PRF;
- A post-operative follow-up of at least 3 weeks;
- The number of patients considered was ≥20;
- The following exclusion criteria were applied;
- The number of patients was <20;
- Case series;
- Case reports;
- A post-operative follow-up of <3 weeks.

### 2.3. Data Extraction

The following data were extracted from the selected studies: the year of publication, the study design, the sample size, the number of OACs treated, the causes of the OAC, the OAC size, the surgical technique used, the number of complications, the success rate of surgery, follow-ups, and the VAS score after surgical treatment.

### 2.4. Quality and Risk-of-Bias Assessment

The OHAT risk-of-bias tool was conducted to assess the risk of bias in the studies included that were classified as a “low risk”, an “unclear risk”, and a “high risk” of bias [25].

The risk-of-bias classes considered for the present analysis were random sequence generation, allocation concealment, a blinding of patients and personnel, a blinding of outcome assessments, an attrition bias, a reporting bias, and other biases.

### 2.5. Data Analysis 

To establish the incidence of the various etiologies, the incidence of every single study was considered. The overall incidence of etiologies was calculated by the amount of the total number of single etiologies. 

### 2.6. Heterogeneity/Meta-Regression 

A high heterogeneity is generally correlated with differences regarding the publication year of the articles included, the different study designs, the healing period, and the population size. The meta-regression computation was conducted through the statistical software package Review Manager (RevMan 5.0, The Nordic Cochrane Centre, The Cochrane Collaboration, Copenhagen, Denmark). For the treatment comparison, studies with similar study designs and an adherence to the inclusion criteria were considered.

### 2.7. Inconsistency Assessment

A node-splitting assessment was performed to detect the inconsistency level of the network meta-analysis. No inconsistency was considered for *p* > 0.05. The level of evidence and network interaction was evaluated with the CINeMA (Confidence in Network Meta-Analysis) Vers. 1 (University of Bern, Germany) system. 

### 2.8. Study Data Analysis

The odds ratio/fixed effects model was applied to assess the significance of the meta-regression analysis and the treatment comparison findings. In full accordance with the Cochrane guidelines, the I2 test considered a low heterogeneity with a value of <40%. For an I2 test with a value of >40%, the heterogeneity was further examined using meta-regressions. The data are provided as mean differences and 95% confidence intervals for the means.

## 3. Results

**Figure 1:**
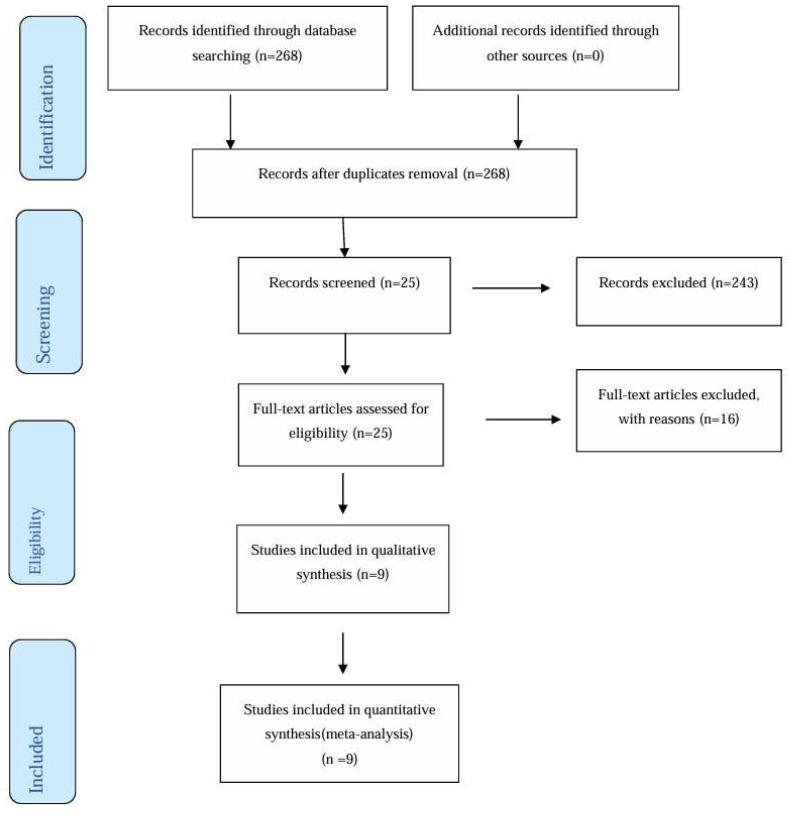
Prisma flow diagram.

The electronic search among the literature yielded 268 articles. After an examination of the titles and abstracts, 25 articles were selected for a full-text review. A total of 16 studies did not meet the inclusion criteria and were excluded. Three articles were excluded because they described a secondary surgery for the treatment of the OAC after the first surgery failed, nine articles were controlled studies with <20 patients, and four studies were excluded because they missed a comparison group. (Bilal; Demetoglu, Gulsen, Bylginair) [26,27,28]. Finally, 9 studies were included for qualitative synthesis (Figure 1).

### 3.1. Study Characteristics

Seven studies among the nine included in this review were randomized controlled trials (RCTs) [29,30,31,32,33,34,35]. Two articles selected were retrospective studies. The minimum follow-up was 3 weeks with a maximum of 1 year. The total number of OACs treated was 519. The frequency of the flap design used for the treatment of the OAC is described in Table 1. The etiologies of the incidences of OACs are summarized in Table 2. The descriptive characteristics of the studies are shown in Table 3. The majority of the studies compared two techniques, only three trials compared three different surgical flaps [8,29,36]. Only one study involved Prf, compared with the buccal advancement flap [35].

### 3.2. Risk of Bias 

**Figure 2:**
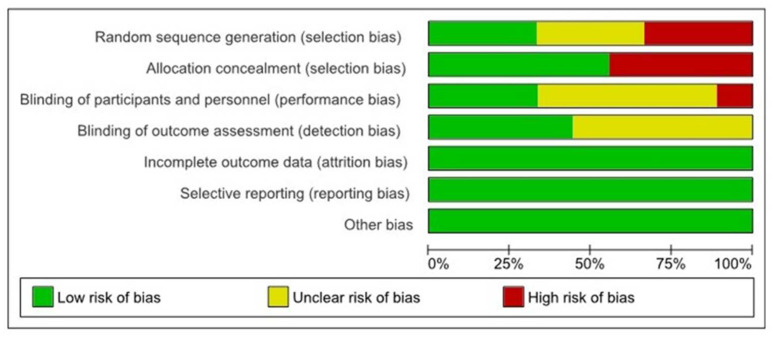
Summary of the OHAT risk-of-bias assessment considering the percentages across all included studies.

**Figure 3:**
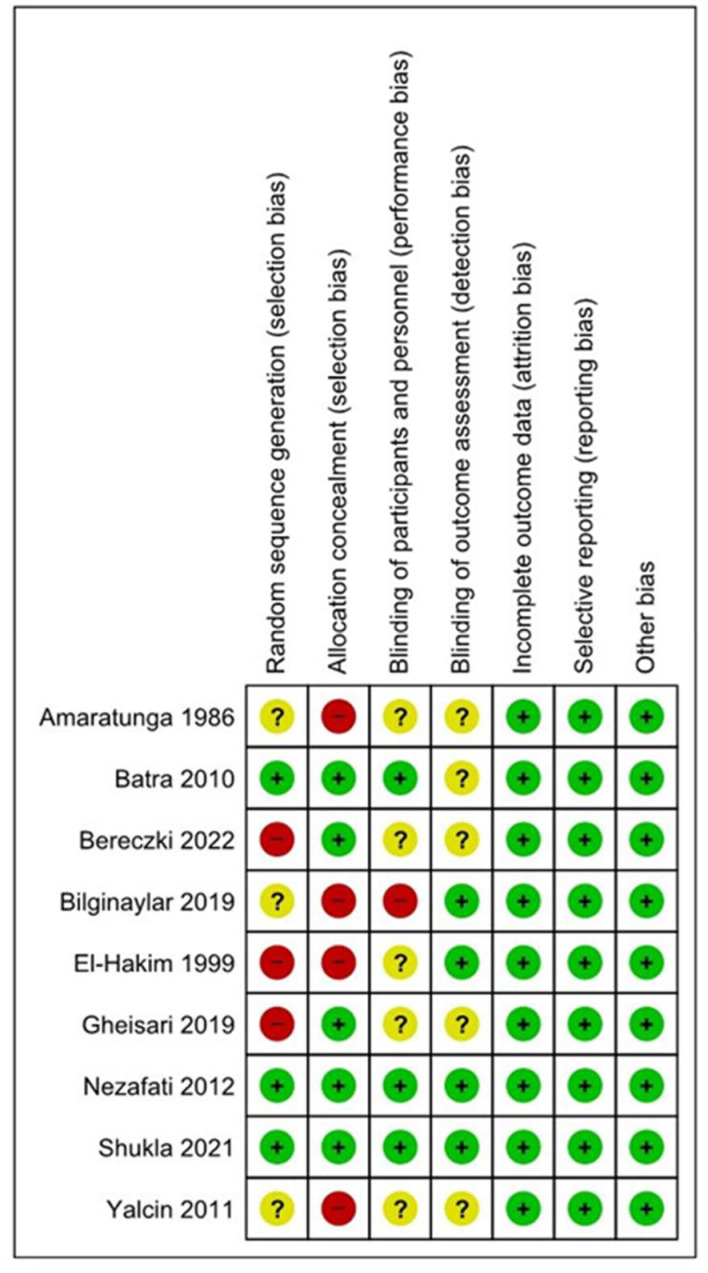
Summary of the OHAT risk-of-bias assessment considering each assessment of the included studies [8,29,30,31,32,33,34,35,36].

The risk-of-bias summary is presented in Figure 2 and Figure 3. A total of 6 studies were considered as having a high risk of bias [8,32,36]. A total of three studies reported a low risk of bias [29,30,31], mainly considering that they did not specify their allocation concealment. There was also a lack of blinding of the participants and personnel [32,35]. A total of three studies had an unclear risk of bias, considering the randomization sequence generator [33,34,35], and three trials had a low risk of bias [32,35].

### 3.3. Comparison of Network Contributions 

**Figure 4:**
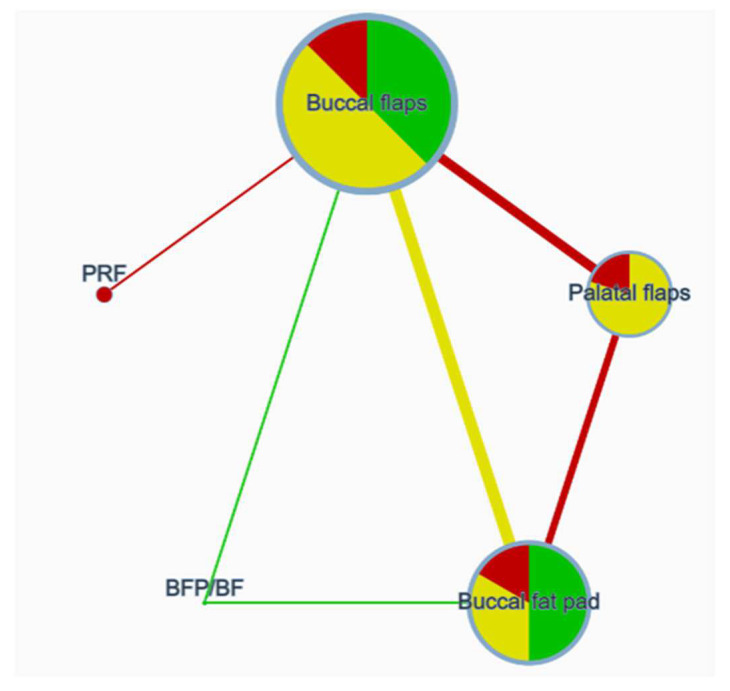
Network plot [node size: sample size; node color: indirectness; edge width: number of studies; edge color: average risk of bias].

**Figure 5:**
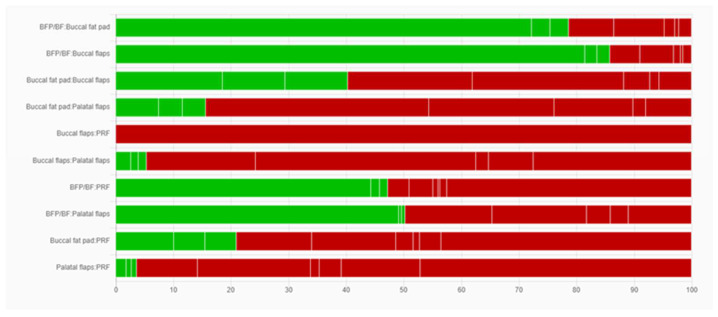
Risk-of-bias contributions. This bar chart shows the contributions of each study to the network estimate.

**Figure 6:**
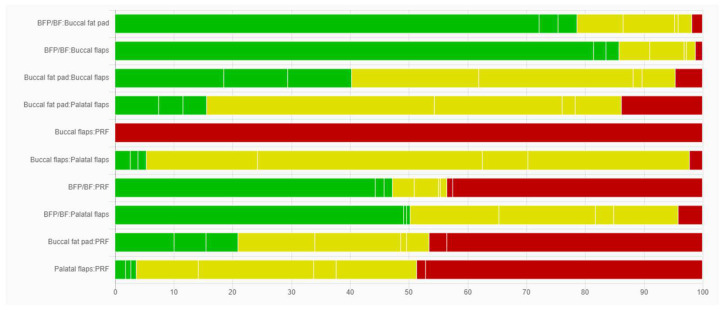
Indirectness contributions. This bar chart shows the contributions of each study to the network estimate.

**Figure 7:**
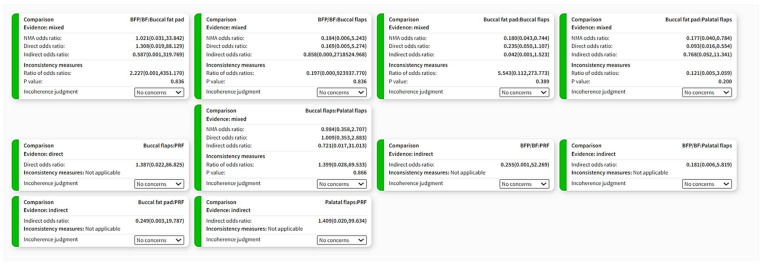
Indirectness contributions. This bar chart shows the contributions of each study to the network estimate.

The network comparison was conducted considering the studies’ sample size, the indirectness detected, the number of studies for each category comparison, and the average risk of bias (Figure 4, Figure 5 and Figure 6). The global test was computed based on a fixed-effects design-by-treatment interaction model and reported a χ^2^ statistic of 1.694 (4 degrees of freedom) and a *p* value of 0.792 (Figure 7). The risk of bias, indirectness, and computation resulted in inconsistencies considering the following treatment comparisons: buccal flaps vs. PRF, palatal flaps vs. PRF, buccal fat pad vs. PRF, buccal fat pad/buccal flap vs. PRF, buccal fat pad/buccal flap vs. palatal flap, and buccal fat pad/buccal flap vs. buccal flap.

### 3.4. Buccal Flap vs. Palatal Flap

**Figure 8:**
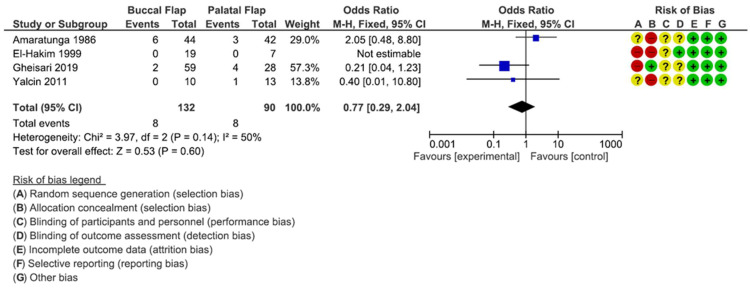
This forest plot shows the comparison between buccal flap vs. palatal flap [32,33,34,36].

The buccal flap/palatal flap comparison is presented in Figure 8.

No significant differences were detected comparing the categories of treatment (*p* = 0.60; Z = 0.53) [OR: −0.77; 95% CI: 0.29; 2.04], with a heterogeneity I2 of 50% [Chi^2^: 3.97; df = 2; *p* = 0.14]. The average risk-of-bias assessment included no studies at a low risk for the comparison of the categories.

### 3.5. Buccal Flap vs. Buccal Fat Pad

**Figure 9:**
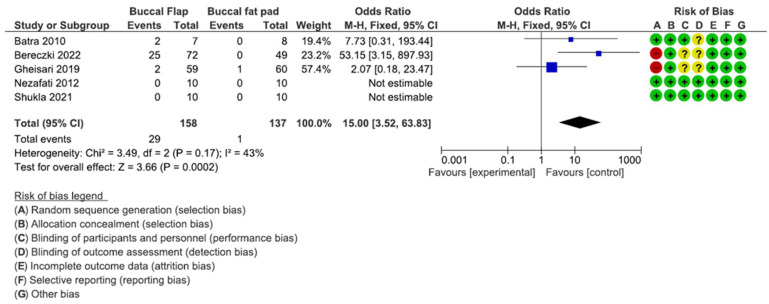
This forest plot shows the comparison between buccal flap vs. buccal fat pad [8,29,30,31,36].

The buccal flap/buccal fat pad comparison is presented in Figure 9. A significant difference was detected in favor of the buccal fat pad (*p* = 0.0002; Z = 3.66) [OR: 15; 95% CI: 3.52; 63.83] with a heterogeneity I2 of 43% [Chi^2^: 3.49; df = 2; *p* = 0.17]. The average risk of bias included 3 studies at a low risk and 2 studies at a high risk.

### 3.6. Palatal Flap vs. Buccal Fat Pad

**Figure 10:**
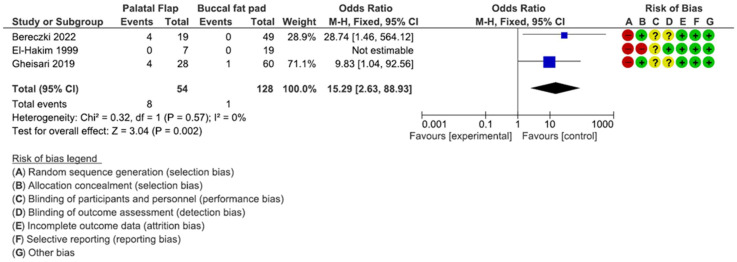
This forest plot shows the comparison between palatal flap vs. buccal fat pad [8,32,36].

The buccal flap/palatal flap comparison is presented in Figure 10. A significant difference was detected in favor of the buccal fat pad (*p* = 0.002; Z = 3.04) [OR: 15.29%; CI: 2.63; 88.93] with a heterogeneity I2 of 0% [Chi^2^: 0.32; df = 1; *p* = 0.57]. The average risk-of-bias assessment included no studies at a low risk of bias.

## 4. Discussion

A main limitation of the present review is certainly associated with the limited number of articles that were eligible for the statistical analysis. A mixed model inclusion criteria was applied considering also non-randomized trials and retrospective studies. According to Faber et al., no previous study has comprehensively assessed the key methodological components common to all systematic reviews and elements specific to the inclusion of non-randomized studies [37]. It should be noted that the present investigation excluded any form of grey literature to avoid plot asymmetries. However, non-randomized trials, including retrospective studies, are prone to confounding, which could lead to an imbalance in prognostic factors correlated with the outcome. Therefore, this could be considered a potential limitation of this study [37].

Oroantral communications and fistulas are complications of oral and maxillofacial surgeries that may specifically result from an anatomical situation or iatrogenic events. The primary goal of this review was to identify the best surgical procedures for the management of oroantral communications following dental surgery. The main outcome of the treatment of oroantral communications is to eradicate the infection site and achieve a complete closure of the communication with no risk of recurrence. Our work primarily focused on identifying the best surgical options in terms of reducing the risk of relapse, safety, and efficacy and a reduced morbidity for the patient.

There are different treatment options available for oroantral communications depending on factors such as the size and location of the defect, the absence of keratinized tissue, and the presence or absence of adjacent teeth. In a systematic review, we analyzed randomized controlled trials and prospective and retrospective studies that compared two or more surgical techniques for treating OACs/OAFs. Our study confirmed previously found data in the literature regarding the causes and effects of oroantral communications. According to the data extracted from the selected articles, the removal of posterior teeth is the primary reason for an oroantral communication (OAC) with an incidence rate of 76%. The second most common cause is cystectomy, accounting for 12%, followed by failed implant positioning at 6%. The buccal advancement flap is the most frequently used technique for treating OACs, with a utilization rate of 43.7%. This technique was first described by Rehrman in 1936 and is still in use today. However, one of its disadvantages is that it can cause a significant decrease in the vestibule due to the coronal positioning of the mucogingival line, which may necessitate vestibuloplasty to increase the width of the keratinized tissue. Buccal edema and postoperative pain are also potential complications associated with this technique. In most cases, the buccal sulcus returns to its original form within 4–8 weeks, but up to 40% of patients may experience lifelong vestibular loss. The vestibular flap technique is effective in treating small- and medium-sized oral communication issues. It is a simple and versatile flap, but it is not suitable for defects that have moved to the palatal region. In such cases, greater flap sliding and vestibular depth loss are required.

After undergoing Rehrmann plasty, it may be necessary to perform an apical reposition flap or an apical reposition flap paired with a free gingival graft (FGG) to improve the width of the keratinized mucosa. The palatal flap is mobilized through a full-thickness incision including the palatal artery to ensure good irroration of the flap. However, the mobility of the flap is limited, which leads to a denudation of the palatal bone, pain, and later roughening and deepening of the area due to secondary epithelialization. In our review, a palatal flap was used in 22% of the cases. It can be used successfully to treat small or medium defects close to the palatal area. The buccal advancement flap and the palatal flap showed no statistically significant difference in terms of the success rate and the risk of relapse. The position of the OAC would be crucial for surgeons’ choices. 

In our analysis, we found that the buccal fat pad (BFP) was commonly used in about 30% of the cases for the closure of an OAC with the lowest rate of relapse. However, the use of BFP is highly dependent on the skills of the operator. Although it has a high success rate, BFP has been associated with various complications such as hematomas and edemas, infections, partial necrosis, large hemorrhages, limiting the mouth opening, excessive scarring, and facial nerve injuries. One of the most significant problems associated with BFP is limiting the mouth opening. Regarding the buccal fat pad-covering technique, the authors described a procedure that consists of a flap repositioning with a partial coverage of the buccal fat pad flap [8,29,30,31,32,36]. The buccal fat pad can be useful in many different surgical occurrences due to the rich vascularization that is able to guide the flap healing and re-epithelialization [19]. 

Complications of BFP included a partial or complete necrosis of adipose tissue, changes in facial contours, postoperative facial edemas, and sometimes facial fistulas. 

In the last few years, several authors described the use of PRF as a membrane sutured to the gingiva to treat OACs [38].

The use of platelet-rich fibrin (PRF) in the treatment of oroantral communications/fistulas (OACs/OAFs) has several advantages. It helps maintain the position of the mucogingival junction without requiring the coronal displacement of mucoperiosteal flaps. This, in turn, prevents associated bone loss and reduces postoperative morbidity. The combined technique of PRF and mucogingival surgery could be a promising approach for further intervention treatments, including dental implants. The balance of the mucogingival line is considered a key factor for dental implant rehabilitation and prosthetic maintenance. In some cases, repositioning with/without a connective graft may be required. The combination of bone grafts still requires a higher level of attention in the literature. Several authors limit this procedure to close 3–4 mm OACs without a purulent discharge and no foreign body within the maxillary sinus antrum [13].

There was only one article, which was a randomized controlled trial (RCT), that compared the use of platelet-rich fibrin (PRF) with the buccal advancement flap for treating oroantral communications (OACs). All other studies that used PRF for treating OACs were either case reports or case series and hence were not included in this review. 

PRF was found to speed up the healing of both soft and hard tissues and also stimulates vascularization and neo angiogenesis. Additionally, PRF has a local immunomodulatory effect that helps regulate inflammation and reduces postoperative morbidity [39].

In order to evaluate the use of PRF in the treatment of OACs, more RCT studies will be needed.

## 5. Conclusions

Performing a comprehensive clinical and radiographic assessment, as well as a detailed review of the patient’s medical history, is crucial to assess the severity of an oroantral communication (OAC) and to decide on the appropriate treatment plan for a patient. The main objective of treating OACs/OAFs is to close the defect and prevent oral bacteria and food particles from entering the sinus. The choice of flap type is determined by several factors, including the size of the wound, the location of the area to be covered, the vascularity of the surrounding area, the specific needs of the patient, and the treatment that will follow wound closure. The amount and quality of surrounding tissues and the possible location of future dental implants also play a role. Additionally, intended results, such as selecting a bone or replacement grafting method, should be considered in cases where dental implant placements will soon be required [21].

Based on the above considerations, we conclude the following:

- The oral and maxillofacial surgeon who treats patients with OACs/OAFs must be aware of and knowledgeable about the numerous treatment options.
- Local soft tissue flaps are a successful modality to treat OACs/OAFs.
- The size and localization of the defect could guide the surgeon in choosing the type of treatment.
- BFP could be the gold standard surgical approach to treat OACs/OAFs because of its lower risk of recurrence.
- The follow-up treatment after communication closure should also be considered.

Due to the lack of high-quality evidence, results must be interpreted with caution. 

## Figures and Tables

**Table 1:** Surgical treatment approach of the studies included.

Buccal flap	227	44%
Buccal fat pad	156	30.00%
Palatal flap	115	22.1%
Prf	21	4%

**Table 2:** Oroantral communication etiology.

Dental extraction	203	76.30%
Failed implants	16	6%
Cystectomy	32	12%
Sinus lift	6	2.20%
Tumor resection	6	2.20%
Endodontic therapy	2	0.70%
Osteonecrosis	1	0.30%

**Table 3:** Descriptive characteristics of studies included for surgical treatment of OACs.

Authors	Year	Type of Study	OAC/OAF Size	Surgical Method	Total Patients	Success (%)	Complication (s)	Follow-Up	VAS Score
Gheisari et al. [36]	2019	Retrospective	5–10 mm	Buccal flaps	(59)	89.8%	2 dehiscence	3 weeks	
				Palatal flaps	(28)	85.7%	4 dehiscence		
				Buccal fat pad	(60)	98.3%	1 dehiscence		
Batra et al. [29]	2010	RCT	5–10 mm	Buccal flap	(7)	72%	2 dehiscence	3 months	
				Buccal fat pad	(8)	100%	-		
				BFP + BF	(6)	100%	-		
Bereczki et al. [8]	2022	Retrospective	3–15 mm	Buccal flap	(72)	73%	25 dehiscence	6 months	
				Buccal fat pad	(49)	100%	-		
				Palatal flap	(19)	75%	4 dehiscence		
Shukla et al. [31]	2021	RCT		Buccal flap	(10)	100%	-	3 weeks	2.9
				Buccal fat pad	(10)	100%			3.5
Nezafati et al. [30]	2012	RCT	6–8 mm	Buccal flap	(10)	100%	-	1 month	2.5
				Buccal fat pad	(10)	100%			4.2
El-Hakim et al. [32]	1999	RCT		Buccal fat pad	(19)	100%	-	12 months	
				Palatal flap	(7)	100%			
Yalcin et al. [33]	2011	RCT		Buccal flap	(10)	100%		6 months	
				Palatal flap	(13)	92%	1 dehiscence		
Amaratunga [34]	1986	RCT		Buccal flap	(44)	86%	6 dehiscence	1 month	
				Palatal flap	(42)	93%	3 dehiscence		
Bilginaylar [35]	2019	RCT	>3 mm	PRF	(21)	100%	-	3 weeks	6
				Buccal flap	(15)	100%			1.4

## Data Availability

All experimental data to support the findings of this study are available contacting the corresponding author upon request.
